# Supplementary material for: Design of Clofazimine-Loaded Lipid Nanoparticles Using Smart Pharmaceutical Technology Approaches
Source: Pharmaceutics. 2025 Jul 2;17(7):873. doi: 10.3390/pharmaceutics17070873 (PMC12300866; doi:10.3390/pharmaceutics17070873)
Supplement: Supplementary file 1 [file pharmaceutics-17-00873-s001.zip › pharmaceutics-3649991-supplementary.pdf]

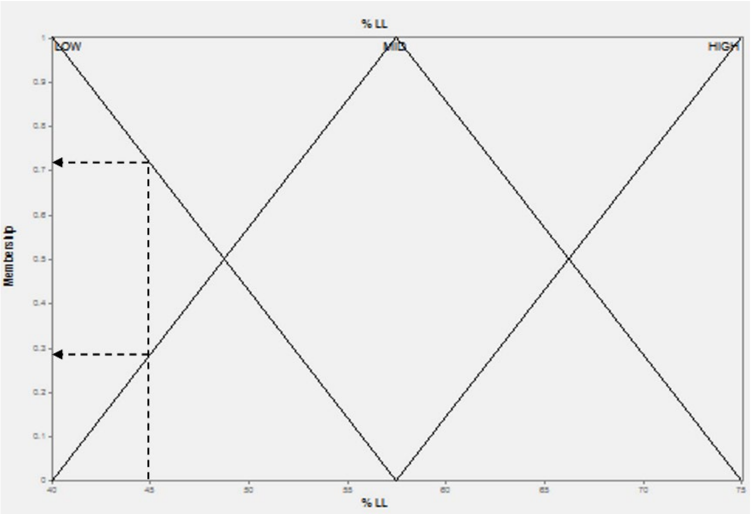

Figure S1. Example of categorization by FormRules® software for the liquid lipid (LL) input, as low, mid, or high, including the associated degree of membership.

**Rules for Size**		
Submodel 1		
IF Lecithin% is LOW	THEN Size (nm) is	LOW (0.83)
IF Lecithin% is MID	THEN Size (nm) is	HIGH (0.92)
IF Lecithin% is HIGH	THEN Size (nm) is	LOW (0.95)
Submodel 2		
IF LL% is LOW	THEN Size (nm) is	LOW (0.64)
IF LL% is MID	THEN Size (nm) is	HIGH (0.88)
IF LL% is HIGH	THEN Size (nm) is	LOW (1.00)

Table S1. Set of IF-THEN rules obtained from NFL models for size. Degree of membership is in parentheses.

**Rules for PdI**		
Submodel 1		
IF Tween 80% is LOW AND Lecithin% is LOW	THEN PdI is	HIGH (1.00)
IF Tween 80% is LOW AND Lecithin% is HIGH	THEN PdI is	LOW (0.75)
IF Tween 80% is HIGH AND Lecithin% is LOW	THEN PdI is	LOW (1.00)
IF Tween 80% is HIGH AND Lecithin% is HIGH	THEN PdI is	HIGH (1.00)
Submodel 2		
IF CFZ% is LOW AND Lecithin% is LOW	THEN PdI is	LOW (1.00)
IF CFZ% is LOW AND Lecithin% is HIGH	THEN PdI is	HIGH (1.00)
IF CFZ% is HIGH AND Lecithin% is LOW	THEN PdI is	HIGH (1.00)
IF CFZ% is HIGH AND Lecithin% is HIGH	THEN PdI is	LOW (1.00)
Submodel 3		
IF Speed is LOW AND LL% is LOW	THEN PdI is	HIGH (0.58)
IF Speed is LOW AND LL% is HIGH	THEN PdI is	LOW (1.00)
IF Speed is HIGH AND LL% is LOW	THEN PdI is	LOW (0.61)
IF Speed is HIGH AND LL% is HIGH	THEN PdI is	HIGH (1.00)

Table S2. Set of IF-THEN rules obtained from NFL models for PdI. Degree of membership is in parentheses.

**Rules for ZP**		
Submodel 1		
IF Tween 80% is LOW AND Lecithin% is LOW	THEN ZP is	LOW (0.84)
IF Tween 80% is LOW AND Lecithin% is HIGH	THEN ZP is	LOW (1.00)

IF Tween 80% is HIGH AND Lecithin% is LOW	THEN ZP is	HIGH (0.70)
IF Tween 80% is HIGH AND Lecithin% is HIGH	THEN ZP is	HIGH (1.00)
Submodel 2		
IF LL% is LOW	THEN ZP is	HIGH (1.00)
IF LL% is HIGH	THEN ZP is	LOW (0.79)

Table S3. Set of IF-THEN rules obtained from NFL models for ZP. Degree of membership is in parentheses.

**Rules for EE%**		
Submodel 1		
IF Lecithin% is LOW AND Speed is LOW	THEN EE% is	LOW (0.63)
IF Lecithin% is LOW AND Speed is HIGH	THEN EE% is	LOW (1.00)
IF Lecithin% is MID AND Speed is LOW	THEN EE% is	LOW (1.00)
IF Lecithin% is MID AND Speed is HIGH	THEN EE% is	HIGH (1.00)
IF Lecithin% is HIGH AND Speed is LOW	THEN EE% is	HIGH (0.90)
IF Lecithin% is HIGH AND Speed is HIGH	THEN EE% is	HIGH (0.58)
Submodel 2		
IF LL% is LOW	THEN EE% is	HIGH (0.87)
IF LL% is MID	THEN EE% is	LOW (0.76)
IF LL% is HIGH	THEN EE% is	HIGH (0.71)
Submodel 3		
IF Tween 80% is LOW	THEN EE% is	HIGH (0.93)
IF Tween 80% is HIGH	THEN EE% is	HIGH (0.9)

Table S4. Set of IF-THEN rules obtained from NFL models for EE%. Degree of membership is in parentheses.

**Rules for DL%**		
Submodel 1		
IF CFZ% is LOW	THEN DL% is	LOW (1.00)
IF CFZ% is MID	THEN DL% is	LOW (0.75)
IF CFZ% is HIGH	THEN DL% is	HIGH (1.00)
Submodel 2		
IF Speed is LOW	THEN DL% is	LOW (0.56)
IF Speed is MID	THEN DL% is	HIGH (0.62)
IF Speed is HIGH	THEN DL% is	LOW (0.72)

Table S5. Set of IF-THEN rules obtained from NFL models for DL%. Degree of membership is in parentheses.

It should be considered that blue colour indicates the combination of inputs that led to the highest value of the output, while red colour shows the combination of inputs giving the lowest value.

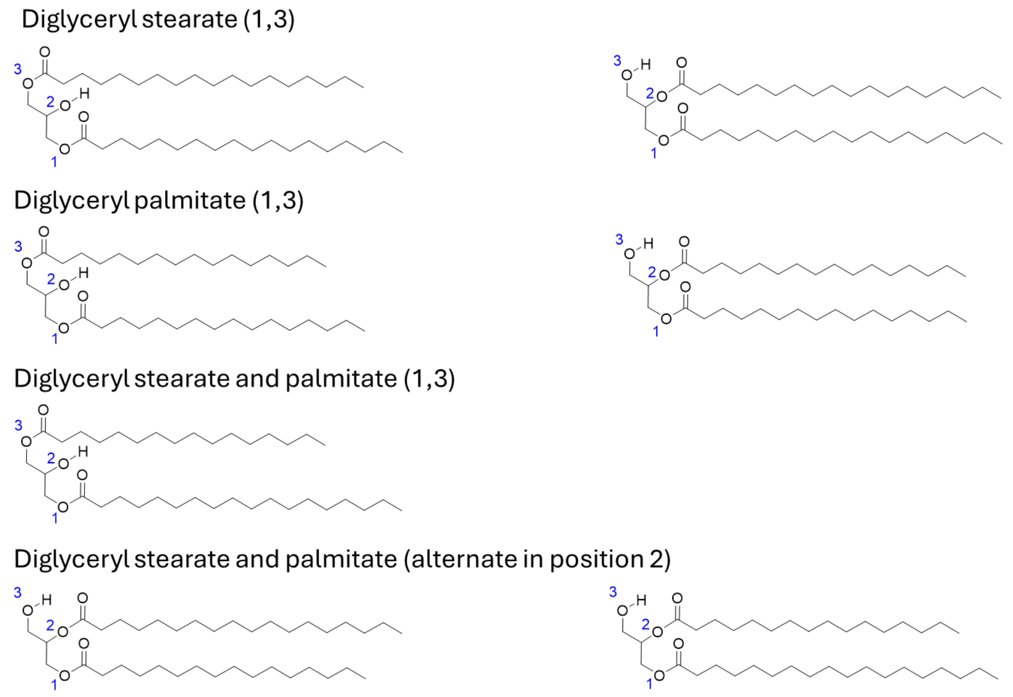

Figure S2. Precirol® ATO 5 glycerolipid components structure.

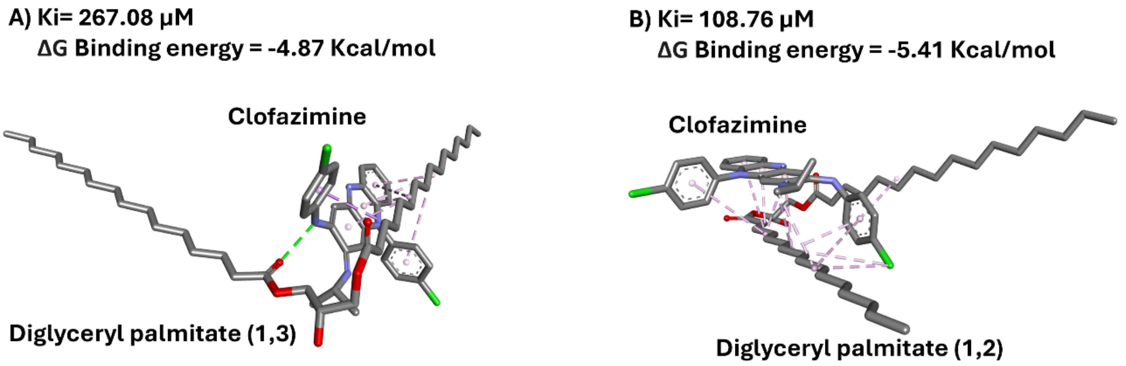

Figure S3. Clofazimine/Diglyceryl palmitate **A)** 1,3 and **B)** 1,2 conformation) binding poses. In the structures, carbon atoms are reported in grey, oxygen atoms in red, nitrogen atoms in blue, and chlorine atoms in green.

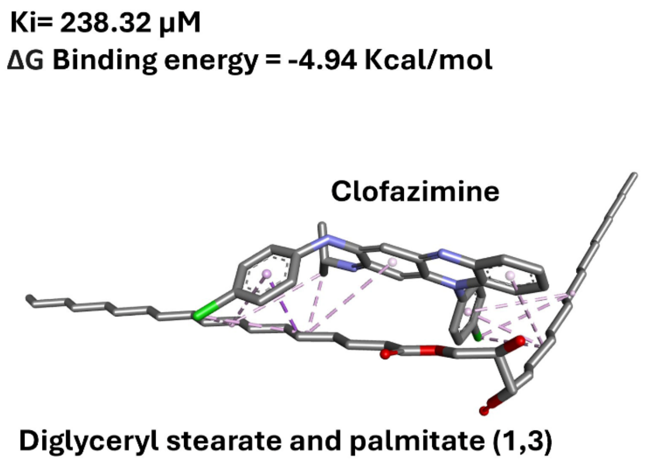

Figure S4. Clofazimine/Diglyceryl stearate palmitate (1,3 conformation) binding pose. Carbon atoms are depicted in grey, oxygen atoms in red, nitrogen atoms in blue, and chlorine atoms in green.

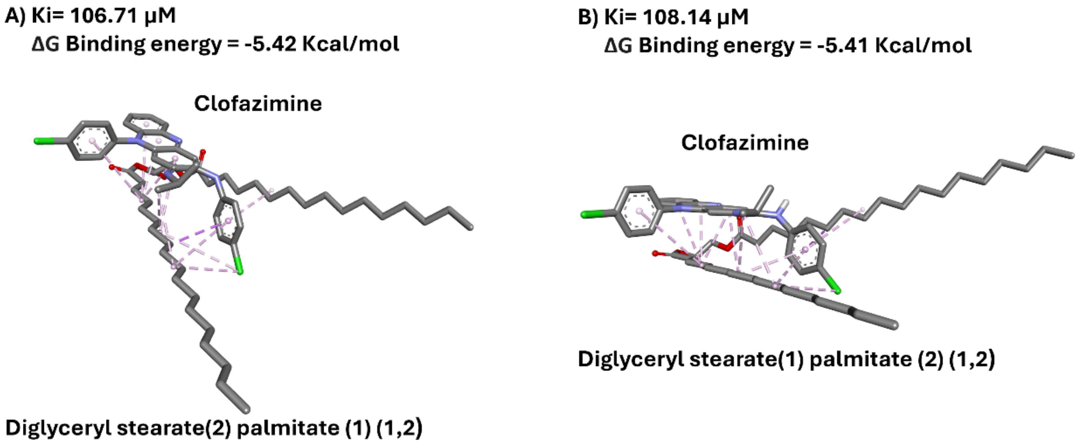

Figure S5. Clofazimine/Diglyceryl stearate palmitate (1,2 conformations) binding poses. Carbon atoms are displayed in grey, oxygen atoms in red, nitrogen atoms in blue, and chlorine atoms in green.

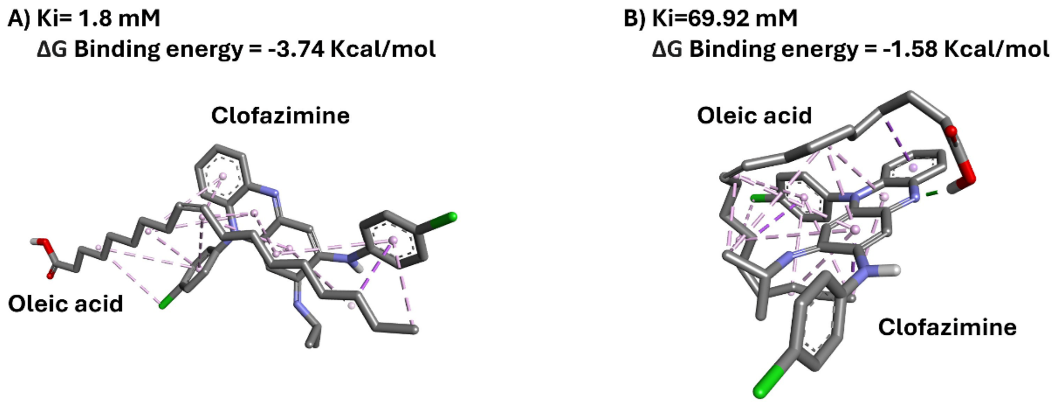

Figure S6. Clofazimine/oleic acid binding poses. In **A)** Oleic acid was used as receptor, while Clofazimine as ligand; in **B)** Clofazimine was used as receptor, while Oleic acid as ligand. Carbon atoms are displayed in grey, oxygen atoms in red, nitrogen atoms in blue, and chlorine atoms in green.
